# Supplementary material for: Quantum biochemical analysis of the TtgR regulator and effectors
Source: Sci Rep. 2024 Apr 12;14:8519. doi: 10.1038/s41598-024-58441-9 (PMC11015042; doi:10.1038/s41598-024-58441-9)
Supplement: Supplementary file 1 — Supplementary Information. [file 41598_2024_58441_MOESM1_ESM.pdf]

Supporting Information for Quantum Biochemical Analysis of the TtgR Regulator and Effectors

E. G. de Carvalho Matias[1], K. S. Bezerra[1], A. H. Lima Costa[1], W. S. Clemente Junior[1], J. I. N. Oliveira[1], L. A. Ribeiro Júnior*[2], D. S. Galvão[3] and U. L. Fulco[1]

[1]*Departamento de Biofísica e Farmacologia, Universidade Federal do Rio Grande do Norte, 59072-970, Natal-RN, Brazil.*

[2]*Institute of Physics, University of Brasília, Brasília, 70919-970, Brazil.*

[3]*Applied Physics Department, University of Campinas, Campinas, São Paulo, Brazil.*

*E-mail: ribeirojr@unb.br

Table S1: Description of TtgR residues interacting with the quercetin effector (QUE) identified in the radius of the binding pocket ranging from 2.0 to 13.0Å. We also expose the regions and groups where there is interaction between TtgR residues and the energetic values (in kcal/mol) for ε=10 and ε=40 calculated by the B97D functional combined with the base set 6-311+G(d.p)

TtgR-QUE Complex

Residue	Atomic Group	Radius (Å)	Energy (ε=10)	Energy (ε=40)
ASP172	i(O4)H	2.0	6.00	4.23
MET89	i(C6)O	2.5	-0.72	-0.62
LEU93	i(C4)H	2.5	-3.16	-2.82
ILE141	i(C2)O	2.5	-4.35	-2.83
VAL171	i(C5)H	2.5	-4.53	-3.92
ILE175	iii(C18)H	2.5	-1.34	-1.16
LEU92	i(O30)H	3.0	-2.09	-1.78
VAL96	iii(C19)H	3.0	-2.38	-2.09
HIS114	iii(C17)O	3.0	-0.03	-0.21
MET167	i(C6)O	3.0	-3.33	-2.51
PHE168	iii(O4)H	3.0	-4.72	-4.65
ASN110	iii(C18)H	3.5	-0.95	-0.89
HIS70	ii(O27)H	4.0	-0.41	-0.44
LEU100	iii(C18)H	4.0	-0.57	-0.46
CYS137	i(C2)O	4.0	-1.53	-1.85
LEU199	i(C6)O	4.0	-0.36	-0.38
ARG176	iii(O4)H	4.5	-2.54	-0.85
LEU202	i(C6)O	4.5	-0.39	-0.25
HIS67	ii(O27)H	5.0	0.14	-0.09
SER77	i(O30)H	5.0	-0.14	-0.10
VAL134	iii(O4)H	5.0	-0.02	-0.23
TYR170	ii(C5)H	5.0	-1.05	-0.61
LEU66	iii(C18)H	5.5	-0.19	-0.19
ALA74	i(O30)H	5.5	-0.09	-0.12
ALA144	i(C1)H	5.5	-0.59	-0.25
LEU145	i(C6)O	5.5	-0.35	-0.22
ALA169	i(C5)H	5.5	-0.22	-0.21
GLY173	iii(O4)H	5.5	-0.42	-0.28
GLU78	i(O30)H	6.0	3.42	0.79
PHE97	iii(C19)H	6.0	-0.48	-0.41
LEU113	iii(C17)O	6.0	-0.29	-0.20
ARG130	iii(O24)H	6.0	-2.09	-0.62
GLY140	i(C2)O	6.0	-0.72	-0.39
ALA164	i(C5)H	6.0	0.41	-0.03
LEU86	i(C1)H	6.5	0.19	0.00
ALA166	i(C5)H	6.5	-0.45	-0.29
LEU179	iii(C17)O	6.5	-0.17	-0.10

GLY198	i(C6)O	6.5	-0.25	-0.11
CYS88	i(O30)H	7.0	-0.24	-0.13
ARG90	i(C1)H	7.0	-2.32	-0.62
THR106	iii(C18)H	7.0	-0.06	-0.04
HIS138	i(C2)O	7.0	-0.02	-0.10
ALA163	i(C6)O	7.0	0.18	0.01
LEU174	iii(O4)H	7.0	-0.47	-0.25
VAL195	i(C6)O	7.0	0.26	0.00
LEU63	iii(O24)H	7.5	0.05	-0.04
LEU73	i(O30)H	7.5	-0.15	-0.08
THR142	i(C1)H	7.5	0.16	0.07
VAL165	i(C5)H	7.5	0.22	0.00
MET201	i(C6)O	8.0	-0.45	-0.14
ASP71	ii(C9)O	8.5	2.27	0.55
ARG75	i(O30)H	8.5	-2.12	-0.54
PRO85	i(C1)H	8.5	0.13	0.02
LYS91	i(O30)H	8.5	-2.45	-0.66
LEU94	i(C6)O	8.5	0.04	0.01
GLN95	iii(C19)H	8.5	-0.21	-0.11
GLU99	iii(C19)H	8.5	2.30	0.55
ARG107	iii(C18)H	8.5	-1.57	-0.40
ILE109	iii(C18)H	8.5	-0.13	-0.07
GLU111	iii(O24)H	8.5	1.68	0.40
ALA133	iii(O24)H	8.5	0.10	-0.01
ASP136	i(O30)H	8.5	2.14	0.46
LYS139	i(O30)H	8.5	-2.39	-0.63
LEU143	i(O30)H	8.5	-0.22	-0.08
ARG177	iii(O24)H	8.5	-1.88	-0.49
LEU187	iii(C19)H	8.5	0.03	-0.01
LEU154	i(C1)H	9	-0.05	-0.02
TRP178	iii(C18)H	9	-0.12	-0.06
TRP194	i(C5)H	9	0.03	-0.01
GLU68	ii(O27)H	9.5	1.73	0.42
THR69	ii(O27)H	9.5	-0.18	-0.06
ALA76	i(O30)H	9.5	-0.14	-0.04
ASN98	iii(C19)H	9.5	-0.09	-0.02
HIS115	iii(O24)H	9.5	-0.01	-0.01
PHE119	iii(O24)H	9.5	0.00	-0.01
LEU135	iii(O4)H	9.5	0.10	0.00
ALA148	i(C1)H	9.5	-0.16	-0.05
ASP196	i(C6)O	9.5	1.34	0.66
ASP200	i(C6)O	9.5	1.12	0.58
HIS72	i(O30)H	10	-0.11	-0.04
GLY87	i(C1)H	10	0.07	0.01

VAL101	iii(C18)H	10	-0.11	-0.04
ILE112	iii(O24)H	10	-0.08	-0.03
GLN131	iii(O4)H	10	0.10	0.02
ALA146	i(C1)H	10	-0.05	-0.02
ALA160	i(C1)H	10	0.25	0.06
THR197	i(C6)O	10	-0.23	-0.07
LEU208	i(C6)O	10	0.03	0.00
ASP64	ii(O27)H	10.5	0.99	0.54
SER65	ii(O27)H	10.5	-0.13	-0.04
ARG162	i(O29)	10.5	-1.88	-0.48
LEU62	iii(C18)H	11	0.03	0.00
SER79	i(O30)H	11	0.13	0.03
ARG108	iii(C18)H	11	-1.37	-0.35
LYS116	iii(O4)H	11	-1.54	-0.39
CYS117	iii(O4)H	11	0.02	0.00
LEU158	i(O29)	11	-0.06	-0.02
GLU161	i(O29)	11	2.46	0.61
LEU180	iii(O24)H	11	-0.06	-0.02
VAL191	iii(C19)H	11	0.15	0.03
ARG203	i(O29)	11	-2.21	-0.56
ASP84	i(C1)H	11.5	2.20	0.55
ARG105	iii(C18)H	12	-1.51	-0.38
GLN129	iii(O4)H	12	0.09	0.02
SER132	iii(O4)H	12	0.04	0.00
ASN147	i(C1)H	12	-0.11	-0.03
VAL59	iii(O24)H	12	0.06	0.01
GLU82	i(O30)H	12	1.95	0.49
LEU102	iii(C18)H	12	-0.10	-0.03
ASP103	iii(C18)H	12	1.71	0.42
VAL149	i(C1)H	12	-0.08	-0.02
GLN153	i(C1)H	12	0.10	0.02
VAL185	iii(C18)H	12	-0.10	-0.03
GLU192	i(C6)O	12	2.09	0.52
LYS193	i(C6)O	12	-1.71	-0.43
ILE126	iii(O24)H	12.5	0.08	0.02
ARG127	iii(O24)H	12.5	-139	-0.35
ASP159	i(O29)	12.5	2.07	0.52
GLN60	ii(O27)H	13	0.21	0.05
ALA104	iii(C18)H	13	0.06	0.01
PRO155	i(O29)	13	0.13	0.03
SER205	i(O29)	13	0.03	0.01

Table S2: Description of TtgR residues interacting with the tetracycline effector (TAC) identified in the radius of the binding pocket ranging from 2.0 to 13.0Å. We also expose the regions and groups where there is interaction between TtgR residues and the energetic values (in kcal/mol) for ε=10 and ε=40 calculated by the B97D functional combined with the base set 6-311+G(d.p).

TtgR-TAC Complex

Residue	Atomic Group	Radius (Å)	Energy (ε=10)	Energy (ε=40)
ASN110	iii(C43)H	2.0	-1.85	-0.57
LEU66	iii(N4)H	2.5	-1.24	-0.60
HIS70	i(C62)H	2.5	-3.17	-3.13
LEU92	i(C62)H	2.5	-2.53	-2.51
LEU93	i(C9)H	2.5	-3.18	-2.02
VAL96	ii(C51)H	2.5	-4.78	-3.14
PHE168	iii(C1)O	2.5	0.45	0.33
VAL171	i(C10)OH	2.5	-3.34	-2.91
HIS67	iii(C4)H	3.0	1.68	1.58
MET89	i(C8)H	3.0	-1.16	-1.29
ARG130	iii(N21)H	3.0	-4.41	-1.75
CYS137	i(C6)OH	3.0	-0.64	-0.53
MET167	i(C10)OH	3.5	-1.72	-1.82
THR106	iii(C42)H	3.5	-0.70	-0.82
ALA133	iii(N21)H	3.5	-1.16	-1.12
ILE141	i(C6)OH	4.0	-2.44	-2.31
LEU63	iii(N21)H	4.0	-0.28	-0.86
LEU100	iii(C42)H	4.0	-0.54	-0.52
LEU113	iii(N21)H	4.0	-3.61	-1.81
HIS114	iii(C43)H	4.0	-0.33	-0.68
VAL134	iii(N21)H	4.0	-0.56	-0.73
ASP172	ii(C12)O	4.0	1.90	-0.47
ILE175	iii(C43)H	4.0	-1.73	-1.44
ALA74	i(C62)H	4.5	0.35	-0.32
GLU99	iii(C42)H	4.5	2.26	0.15
ASP64	iii(N21)H	5.5	2.55	0.88
THR69	iii(C42)H	5.5	-0.45	-0.28
ILE109	iii(C43)H	5.5	-0.52	-0.41
LEU199	i(C9)H	5.5	-0.09	-0.11
CYS88	i(C8)H	6.0	-0.09	-0.08
ARG90	i(C8)H	6.0	-1.59	-0.47
GLN95	i(C62)H	6.0	-0.19	-0.21
PHE97	i(C10)OH	6.0	-0.51	-0.52
ALA164	i(C10)OH	6.0	0.13	-0.04
ALA169	i(C10)OH	6.0	0.19	-0.01
LEU202	i(C9)H	6.0	-0.11	-0.08

LEU62	iii(C3(O	6.5	-0.04	-0.07
SER65	iii(C42)H	6.5	-0.87	-0.6
SER77	i(C7)H	6.5	-0.15	-0.09
ARG107	iii(C43)H	6.5	-1.77	-0.50
GLN129	iii(N21)H	6.5	0.02	-0.04
LEU145	i(C9)H	6.5	-0.16	-0.09
TYR170	i(C10)OH	6.5	-0.61	-0.45
GLU68	iii(C42)H	7.0	2.41	0.60
ASP71	i(C62)H	7.0	02.09	0.49
LEU73	i(C7)H	7.0	-0.23	-0.15
LYS91	i(C8)H	7.0	-1.63	-0.46
LEU94	i(C8)H	7.0	-0.03	-0.08
GLU111	iii(C43)H	7.0	2.65	0.71
HIS138	i(C6)OH	7.0	-0.47	-0.26
GLY140	i(C6)OH	7.0	-0.38	-0.18
ALA144	i(C8)H	7.0	-0.22	-0.10
LEU179	iii(C43)H	7.0	-0.20	-0.11
VAL195	i(C9)H	7.0	0.05	-0.04
VAL101	iii(C42)H	7.5	-0.05	-0.04
ARG105	iii(C43)H	7.5	-2.17	-0.58
GLN131	iii(N21)H	7.5	0.12	-0.01
SER132	iii(N21)H	7.5	-0.20	-0.10
ASP136	i(C6)OH	7.5	2.46	0.52
VAL165	i(C10)OH	7.5	0.20	0.01
ALA166	i(C10)OH	7.5	0.03	-0.01
GLY173	ii(C12)O	7.5	-0.05	-0.05
ARG176	iii(C21)O	7.5	-3.16	-0.87
GLY198	i(C10)OH	7.5	-0.10	-0.04
GLN60	iii(N21)H	8.0	0.56	0.12
ARG75	i(C62)H	8.0	-2.41	-0.65
GLU78	i(C62)H	8.0	2.4	0.56
LEU86	i(C8)H	8.0	0.06	-0.01
ASN98	iii(C42)H	8.0	-0.14	-0.07
ARG108	iii(C43)H	8.0	-2.02	-0.53
PHE119	iii(N21)H	8.0	-0.01	-0.02
LEU135	iii(N21)H	8.0	-0.03	-0.05
ALA163	i(C10)OH	8.0	0.07	0.00
LEU187	iii(C43)H	8.0	-0.06	-0.04
VAL59	iii(N21)H	8.5	0.18	0.03
PRO85	i(C8)H	8.5	0.05	0.00
ASP103	iii(C42)H	8.5	02.03	0.49
LEU174	ii(C12)O	8.5	-0.37	-0.17
HIS72	i(C62)H	9.0	-0.09	-0.05
GLY87	i(C8)H	9.0	0.02	0.00

ILE112	iii(C43)H	9.0	-0.26	-0.11
ILE126	iii(N21)H	9.0	0.18	0.04
LYS139	i(C6)OH	9.0	-2.71	-0.72
THR142	i(C6)OH	9.0	-0.17	-0.08
TRP178	iii(C43)H	9.0	-0.15	-0.07
MET201	i(C10)OH	9.0	-0.16	-0.06
ALA61	iii(C3(O	9.5	0.12	0.02
ALA76	i(C62)H	9.5	-0.08	-0.03
LEU102	iii(C42)H	9.5	-0.1	-0.04
ALA104	iii(C43)H	9.5	0.07	0.01
ARG127	iii(N21)H	9.5	-1.82	-0.46
GLN128	iii(N21)H	9.5	0.08	0.01
CYS117	iii(N21)H	10.0	0.03	0.00
LEU154	i(C9)H	10.0	-0.05	-0.02
ARG177	ii(C12)O	10.0	-1.87	-0.48
TRP194	i(C10)OH	10.0	0.02	-0.01
ASP196	i(C9)H	10.0	1.7	0.41
HIS115	iii(C43)H	10.5	-0.06	-0.03
ALA148	i(C8)H	10.5	-0.07	-0.02
ARG162	i(C10)OH	10.5	-1.4	-0.35
GLU21	iii(C42)H	11.0	2.14	0.53
ASP84	i(C8)H	11.0	1.45	0.36
GLU125	iii(N21)H	11.0	1.80	0.45
LEU143	i(C8)H	11.0	-0.14	1.53
GLU161	i(C10)OH	11.0	2.20	0.55
VAL191	i(C9)H	11.0	0.06	0.00
THR197	i(C9)H	11.0	-0.12	-0.04
ASP200	i(C9)H	11.0	1.45	0.35
TYR25	iii(C43)H	11.5	-0.04	-0.01
LEU58	iii(N21)H	11.5	0.10	0.02
ALA146	i(C8)H	11.5	-0.06	-0.02
ASN147	i(C8)H	11.5	-0.16	-0.05
ALA160	i(C9)H	11.5	0.05	0.01
LEU208	i(C9)H	11.5	0.01	0.00
VAL29	iii(N21)H	12.0	-0.09	-0.03
ALA56	iii(N21)H	12.0	0.14	0.04
SER79	i(C7)H	12.0	0.08	0.02
GLU82	i(C8)H	12.0	1.39	0.34
LYS116	iii(N21)H	12.0	-2.11	-0.54
VAL185	ii(C12)O	12.0	0.01	0.00
LEU188	iii(C42)H	12.0	0.04	0.00
ARG203	i(C9)H	12.0	-1.39	-0.35
ILE17	iii(N4)H	12.5	0.03	0.00
PHE24	iii(C3)O	12.5	-0.07	-0.02

GLU57	iii(N21)H	12.5	1.84	0.46
ARG151	i(C8)H	12.5	-1.48	-0.37
GLN153	i(C8)H	12.5	0.03	0.01
LEU180	iii(C43)H	12.5	-0.05	-0.02
GLU192	i(C9)H	12.5	1.58	0.39
LYS193	i(C9)H	12.5	-1.37	-0.34
ARG13	iii(C3)O	13.0	-1.95	-0.49
LEU158	i(C9)H	13.0	0.00	0.00

Table S3: Description of TtgR residues interacting with the chloramphenicol (CLM) effector identified in the radius of the binding pocket ranging from 2.0 to 13.0Å. We also expose the regions and groups where there is interaction between TtgR residues and the energetic values (in kcal/mol) for ε=10 and ε=40 calculated by the B97D functional combined with the base set 6-311+G(d.p)

TtgR-CLM Complex

Residue	Atomic Group	Radius (Å)	Energy (ε=10)	Energy (ε=40)
LEU92	i(C11)H	2.5	-2.47	-2.43
LEU93	ii(C5)H	2.5	-2.07	-2.05
VAL96	ii(C4)H	2.5	-2.20	-1.62
CYS137	i(C8)H	2.5	2.66	2.80
GLY140	i(N9)O	2.5	-1.47	-1.38
ILE141	i(C8)H	2.5	-3.11	-3.06
VAL171	ii(O4)H	2.5	-1.86	-1.79
HIS70	iii(C2)O	3.0	-2.03	-1.86
GLU78	i(N9)O	3.0	1.12	1.31
PHE168	i(C7)H	3.0	-2.64	-2.61
ILE175	ii(C4)H	3.0	-0.96	-0.93
HIS67	iii(C1)Cl	3.5	-2.08	-1.94
ALA74	i(N9)O	3.5	-0.97	-0.91
MET89	i(C11)H	3.5	-1.99	-1.74
PHE97	ii(C4)H	4.0	-0.52	-0.52
SER77	i(C10)H	4.5	-0.40	-0.37
ASP136	i(N9)O	4.5	0.07	-0.27
HIS138	i(C8)H	4.5	0.26	0.28
ASP172	ii(O4)H	4.5	-0.91	-0.63
LEU66	iii(C1)H	5.0	-0.56	-0.58
LYS139	i(N9)O	5.0	-0.92	-0.58
ALA144	i(N9)O	5.0	-0.30	-0.27
ALA133	iii(C1)Cl	5.5	-0.27	-0.23
VAL134	iii(C1)Cl	5.5	-0.34	-0.34
MET167	ii(O5)H	5.5	-0.58	-0.55
ARG75	i(N9)O	6.0	-0.53	-0.26

THR142	i(N9)O	6.0	-0.10	-0.05
GLU68	iii(C1)Cl	6.5	0.13	-1.00
LEU73	i(C10)H	6.5	-0.21	-0.16
LEU143	i(N9)O	6.5	-0.23	-0.16
LEU63	iii(C1)Cl	7.0	-0.19	-0.16
ASP71	iii(C1)Cl	7.0	0.29	0.03
CYS88	i(C10)H	7.0	-0.06	-0.05
LEU100	ii(C4)H	7.0	-0.06	-0.03
ASN110	ii(C4)H	7.0	-0.24	-0.19
LEU135	i(N9)O	7.0	-0.12	-0.11
ALA164	i(C8)H	7.0	-0.14	-0.13
ALA169	ii(O5)H	7.0	-0.12	-0.10
TYR170	ii(O5)H	7.0	-0.13	-0.13
ASP64	iii(C1)Cl	7.5	0.02	-0.04
ARG90	i(C11)H	7.5	-1.00	-0.02
LEU94	ii(C5)H	7.5	-0.12	-0.08
GLN95	ii(C2)O	7.5	-0.08	-0.03
LEU113	iii(C1)H	7.5	-0.05	-0.03
LEU145	i(N9)O	7.5	-0.10	-0.10
GLY173	ii(O4)H	7.5	-0.03	-0.03
LEU174	ii(C4)H	7.5	-0.01	-0.04
LEU199	i(C11)H	7.5	-0.03	-0.04
THR69	iii(C1)Cl	8.0	-0.20	-0.13
ALA76	i(N9)O	8.0	-0.03	-0.03
LYS91	i(C11)H	8.0	-0.17	-0.10
GLU99	iii(C1)H	8.0	-0.18	-0.09
THR106	ii(C4)H	8.0	-0.04	-0.04
HIS114	ii(C4)O	8.0	-0.07	-0.05
ARG130	iii(C1)Cl	8.0	0.15	-0.01
VAL195	ii(C5)H	8.0	-0.05	-0.05
SER65	i(C1)Cl	8.5	-0.01	-0.04
SER79	i(N9)O	8.5	0.06	0.04
PRO85	i(C10)H	8.5	-0.02	-0.02
SER132	iii(C1)Cl	8.5	-0.01	-0.03
VAL165	i(C7)H	8.5	-0.07	-0.06
ALA166	ii(O5)H	8.5	-0.06	-0.04
ARG176	ii(O4)H	8.5	0.18	0.02
LEU187	ii(C4)H	8.5	-0.01	-0.01
LEU202	i(C11)H	8.5	-0.03	-0.03
HIS72	i(N9)O	9.0	-0.02	-0.03
LEU86	i(C10)H	9.0	-0.03	-0.02
ASN98	ii(C4)H	9.0	-0.02	-0.02
ASN147	i(N9)O	9.0	-0.06	-0.03
GLY198	ii(O5)H	9.0	0.01	-0.01

LEU62	iii(C1)H	9.5	-0.01	-0.01
ILE109	iii(C1)H	9.5	0.00	-0.01
ALA163	ii(C5)O	9.5	-0.04	-0.03
LEU179	ii(C4)H	9.5	0.00	-0.01
GLN129	iii(C1)Cl	10.0	0.01	-0.01
GLN131	iii(C1)Cl	10.0	-0.04	-0.02
ALA146	i(N9)O	10.0	-0.04	-0.02
MET201	ii(O5)H	10.0	0.00	-0.01
GLY87	i(C10)H	10.5	-0.04	-0.02
VAL101	ii(C4)H	10.5	-0.01	-0.01
ALA148	i(C10)H	10.5	-0.01	-0.01
TRP178	ii(C4)H	10.5	0.00	-0.01
TRP194	ii(C4)H	10.5	0.00	-0.01
GLU82	i(C10)H	11.0	0.10	0.02
ARG107	ii(C4)H	11.0	0.09	0.02
LEU154	i(C10)H	11.0	0.00	-0.01
ALA160	i(C8)H	11.0	-0.02	-0.01
GLU161	i(C8)H	11.0	0.10	0.02
ARG177	ii(C4)O	11.0	0.13	0.03
VAL191	ii(C4)H	11.0	-0.03	-0.01
GLN60	iii(C1)H	11.5	-0.03	-0.02
ASP84	i(C10)H	11.5	0.01	0.00
ASP196	ii(C5)H	11.5	-0.12	-0.03
ALA61	iii(C1)H	12.0	0.01	0.00
ASP103	ii(C4)H	12.0	-0.11	-0.03
GLU111	ii(C4)H	12.0	-0.17	-0.05
ARG162	ii(C5)O	12.0	-0.01	-0.01
THR197	ii(O5)H	12.0	0.02	0.00
VAL59	iii(C1)H	12.5	-0.01	-0.01
GLU80	i(N9)O	12.5	0.13	0.03
ARG150	i(N9)O	12.5	-0.16	-0.04
VAL185	ii(C4)H	12.5	-0.01	0.00
LEU102	ii(C4)H	13.0	0.00	0.00
ARG105	iii(C1)H	13.0	0.00	0.00
PHE119	iii(C1)Cl	13.0	0.00	0.00
GLN128	iii(C1)Cl	13.0	0.00	0.00
VAL149	i(C10)H	13.0	0.00	0.00
GLN153	i(C10)H	13.0	0.00	0.00
LEU158	i(C10)H	13.0	0.00	0.00
ASP200	ii(O5)H	13.0	0.00	0.00
LEU208	ii(C5)O	13.0	0.00	0.00
